# Identifying factors that nurses consider in the decision-making process related to patient care during the COVID-19 pandemic

**DOI:** 10.1371/journal.pone.0254077

**Published:** 2021-07-02

**Authors:** Nicholas Anton, Tera Hornbeck, Susan Modlin, Md Munirul Haque, Megan Crites, Denny Yu

**Affiliations:** 1 School of Industrial Engineering, Purdue University, West Lafayette, IN, United States of America; 2 School of Nursing, Purdue University, West Lafayette, IN, United States of America; 3 RB Annis School of Engineering, University of Indianapolis, Indianapolis, IN, United States of America; Universitat d’Alacante, SPAIN

## Abstract

**Background:**

Nurse identification of patient deterioration is critical, particularly during the COVID-19 pandemic, as patients can deteriorate quickly. While the literature has shown that nurses rely on intuition to make decisions, there is limited information on what sources of data experienced nurses utilize to inform their intuition. The objectives of this study were to identify sources of data that inform nurse decision-making related to recognition of deteriorating patients, and explore how COVID-19 has impacted nurse decision-making.

**Methods:**

In this qualitative study, experienced nurses voluntarily participated in focused interviews. During focused interviews, expert nurses were asked to share descriptions of memorable patient encounters, and questions were posed to facilitate reflections on thoughts and actions that hindered or helped their decision-making. They were also asked to consider the impact of COVID-19 on nursing and decision-making. Interviews were transcribed verbatim, study team members reviewed transcripts and coded responses, and organized key findings into themes.

**Results:**

Several themes related to decision-making were identified by the research team, including: identifying patient care needs, workload management, and reflecting on missed care opportunities to inform learning. Participants (n = 10) also indicated that COVID-19 presented a number of unique barriers to nurse decision-making.

**Conclusions:**

Findings from this study indicate that experienced nurses utilize several sources of information to inform their intuition. It is apparent that the demands on nurses in response to pandemics are heightened. Decision-making themes drawn from participants’ experiences can to assist nurse educators for training nursing students on decision-making for deteriorating patients and how to manage the potential barriers (e.g., resource constraints, lack of family) associated with caring for patients during these challenging times prior to encountering these issues in the clinical environment. Nurse practice can utilize these findings to increase awareness among experienced nurses on recognizing how pandemic situations can impact to their decision-making capability.

## Introduction

In hospital settings, the failure to identify deteriorating patients can lead to delays in appropriate patient care [1–3], which can cause increased morbidity and mortality [4, 5]. A recent qualitative study of surgical intensive care unit (ICU) nurses found that their ability to quickly identify deteriorating patients led to the early deployment of the rapid response team [6]. Early nurse intervention to manage patient deterioration may be particularly critical in managing patients suffering from the SARS-CoV-2-causing Coronavirus Disease 19 (COVID-19). Patients suffering from COVID-19 can deteriorate quickly requiring rapid decision-making and responses from health care providers [7]. Although it is known that nurses routinely make accurate, life-saving decisions, it is less well known what sources of information nurses utilize in their clinical decision-making.

Investigators have shown that expert nurses possess both an understanding of specific disease processes and a broad holistic understanding of acute patient care situations [8]. Based on their wealth of experience, expert nurses may utilize intuitive or subconscious decision-making processes to quickly grasp complex clinical situations, rapidly and confidently come to an accurate assessment and provide safe quality care to patients [9, 10]. According to Benner et al. (1992), the application of the Dreyfus model of skill acquisition to clinical nursing practice has helped explain how nurse decision-making develops over time [11, 12]. The authors suggest that over time, nurses transition from reliance on abstract principles to use of past experiences to guide performance. Expert nurses shift from reliance on rule-based thinking (e.g., checklists or rigid protocols) to using intuition, therefore the expert nurse is able to attend selectively to details of increasingly complex situations [11]. However, the literature also emphasizes the importance of nurses’ critical thinking in their decision-making processes.

Shoulders et al. (2014) define the nursing process as the thought process used to collect information, assess that information, and solve patient care problems [13]. Upon systematically gathering data, nurses utilize critical thinking to interpret that data and identify task-relevant data to focus on during the assessment process. Nurses may also utilize critical thinking in their analysis of the reliability of important information and as a method to validate initial judgments in the assessment process [14]. It is important to note that according to the Cognitive Continuum Theory, decision-making is executed using a combination of intuition and critical thinking processes and fluctuates depending on the demands of the task (e.g., if adequate time or information is available to afford critical thinking) [15].

While research has shown that experienced nurses rely on a combination of intuition and critical thinking, few studies have attempted to identify what specific factors or sources of data inform experienced nurse decision-making when caring for deteriorating patients. Furthermore, there is a dearth of research on how immense disruptions caused by the onset of global pandemics can affect nurse clinical decision-making. Accordingly, the purposes of this study were to:

1. Identify specific sources of data and factors that inform nurse decision-making when caring for deteriorating patients, and
2. Explore how COVID-19 has impacted nurse decision-making.

The ultimate goal of this qualitative study is to synthesize key aspects of experienced nurse intuition and decision-making that can be disseminated to nurses in training and to provide understanding of how pandemics can influence these processes.

## Materials and methods

In order to achieve the aims of this study, interviews were conducted virtually with experienced nurses. Inclusion criteria for the study consisted of practicing nurses (i.e., no longer in training), who were in clinical practice at the time of interviews (i.e., not full-time academic appointment). Exclusion criteria included nurses still in training or who were non-clinical. In-depth, semi-structured interviews with nurses focused on two areas: 1) decision-making in the context of deteriorating patient recognition, and 2) the impact of COVID-19 on nurse decision-making capabilities. This study was approved by the Institutional Review Board (IRB-2020-11) and met university safety standards for conducting research during COVID-19.

### Participant and site information

Nurses with diverse backgrounds (e.g., ICU, emergency department, etc.) were invited to participate via email. Prior to the interviews, participants were provided with general information about the purpose of the study, specific examples of questions were provided to participants during recruitment, and informed consent was obtained from each participant electronically. The stopping criteria for participant recruitment was when data saturation was reached (i.e., no new information was gathered from the participants) [16]. No repeat interviews were carried out. All participants were interviewed after April 2020.

### Questionnaire—Demographics

Participants provided demographic information using a REDCap survey system including age, gender, nursing and other degrees obtained, years of clinical experience, clinical specialty, bed size of the hospital, and the average number of hours worked per week.

### Focused interviews

Due to COVID-19 restrictions about face-to-face meetings and increased work demands for nurses, we hosted virtual interviews with small groups of 1–3 participants. Regardless of group size, the interviewer deliberately solicited responses to each question from each participant to prevent individuals from dominating group interviews and allow all participants to respond. While we intended to utilize a snowball sampling approach to participant recruitment, the increased work demands for nurses during the COVID-19 pandemic prevented our team from accruing an adequate sample size, as participants’ colleagues were unable to participate due to time constraints. We instead relied on purposive sampling that targeted nurses who were known to the research team and who were experienced (i.e., more than 5 years of clinical experience) and had substantial nursing education. All participants engaged in virtual focused interviews conducted via Webex (Cisco Webex, Cisco Systems, Milpitas, California), which were moderated by an expert qualitative researcher for 60–90 minutes. Aside from participants and researchers, no one else was present during the focused interviews.

Each focused interview began with introductions and an overview of the purpose of the study. At this time, the interviewer shared details about her role at the university as faculty and her research interests in decision-making. Participants were then asked to share a description of their most memorable patient encounter. Additional questions were then posed to elicit clinical examples of a patient who was stable and deteriorated or a patient who was unstable but returned to being stable. Prompts were utilized to facilitate reflection on their thoughts and actions that helped or hindered their decision-making. The first two interviews our team conducted occurred in April of 2020, and the COVID-19 pandemic had already significantly affected healthcare systems in the United States. During these interviews, both participants discussed the impact of COVID-19 on nursing practice. Accordingly, our team felt questions about how nurse decision-making had been affected by the pandemic were important to include in our study. In all remaining interviews, nurses were asked to consider the impact of COVID-19 on nursing and their decision-making capabilities. The finalized interview guide can be found in S1 Appendix.

### Data analysis

All interviews were audio-recorded, and field notes were taken during and after each interview. Recordings were de-identified and professionally transcribed verbatim. Transcriptions and findings were not made available to participants for comment or correction, as we aimed to maintain a robust data set and avoid artificially deriving consensus among participant responses if agreement was not obtained naturally. Utilizing a qualitative description approach [17], and an inductive content analysis process, each interview transcription was reviewed by study team members (i.e., including clinical subject matter experts and human factors engineers) individually and collectively after each interview. Qualitative description is a process of discovering and understanding phenomena or perspectives from a target population [18]. Qualitative description is particularly useful in healthcare-based studies, as this approach seeks to understand the perspective of those experiencing key phenomena and provides a direct description of the phenomena according to participants.

In the present study, each transcript was reviewed carefully and key themes were highlighted and assigned a code. Transcripts were then carefully reviewed again and meaningful codes were grouped into themes and sub-themes. This process was iterative, as themes were refined further or removed upon subsequent analyses of additional transcripts. NVivo 12 (QSR International, Melbourne, Australia) was used as a data repository to sort codes, themes, and complete an analysis of participants’ responses based on common words to identify which participants used similar terminology [19, 20].

In order to increase the rigor of our findings, our team utilized a triangulation approach to our analysis (i.e., as defined by Lincoln & Guba, 1985) [21]. Triangulation among our interdisciplinary study team included regular virtual meetings to reach consensus about a coding framework, to aggregate codes into themes, and identify sub-themes. Data saturation was reached with 10 participants, as no new themes emerged from the final interview.

## Results

**Fig 1:**
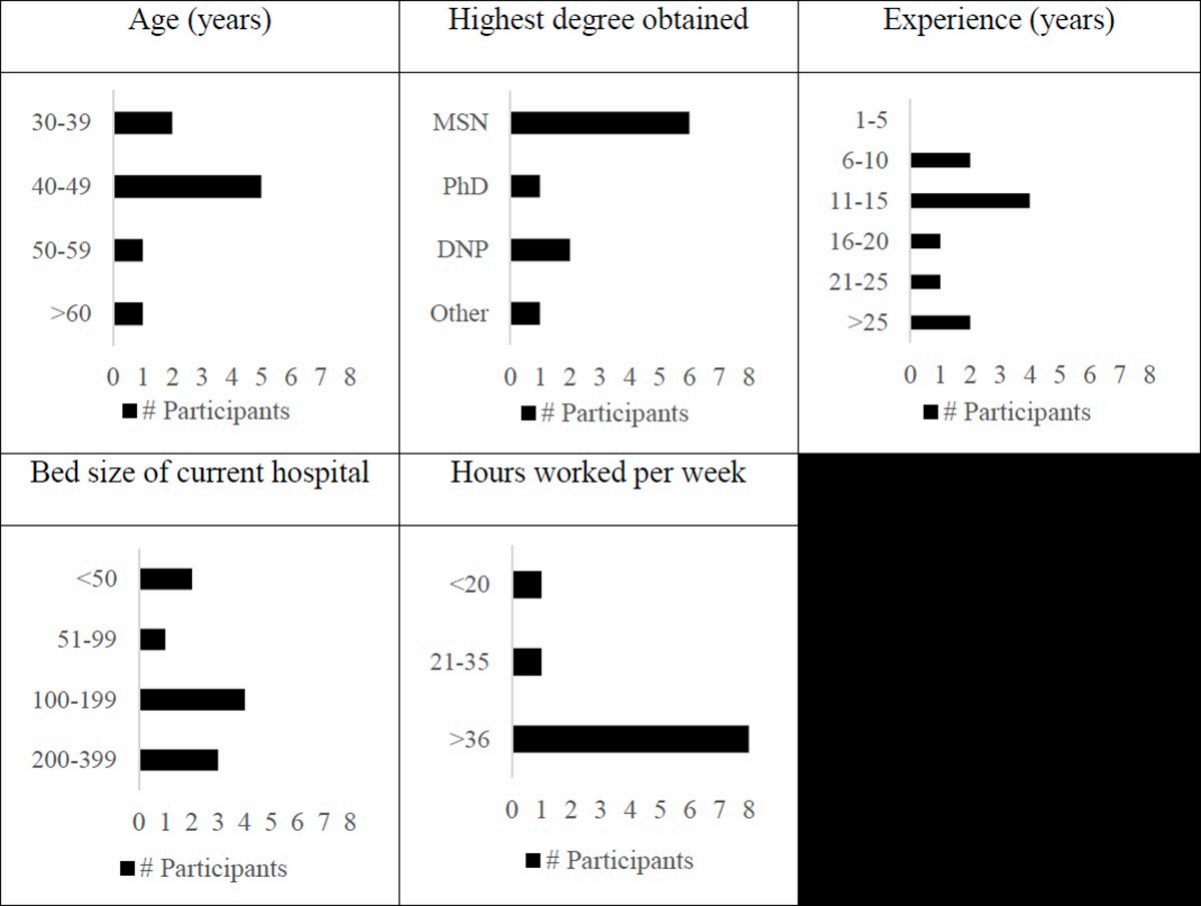
Demographic information collected from participants (note that one participant did not provide age).

A total of 10 nurses (100% females) participated in the focused interviews (Fig 1). No prospective participants refused to participate or dropped out from the study. Units where nurses spend the majority of their time were: primary care (n = 1), intensive care unit (n = 3), palliative care (n = 1), long-term care (n = 2), medical-surgical acute care(n = 2), and risk analysis (n = 1).

### Factors impacting nurse decision-making

**Fig 2:**
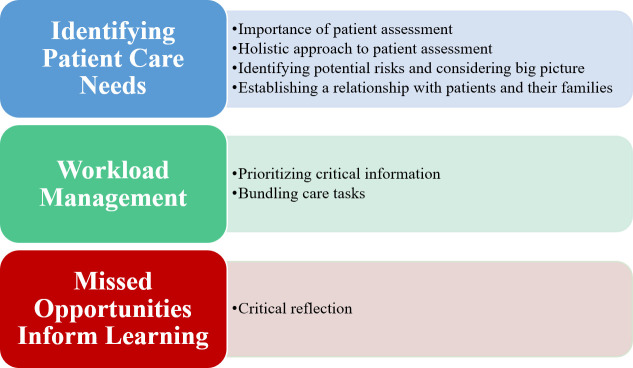
Interview themes and subthemes.

Findings from the focused interviews established fundamental components of nurse decision-making, and also emphasized the complex tradeoffs between systems-level barriers (e.g., available resources) and the provision of safe and quality care to patients. Themes and subthemes are presented in Fig 2.

#### Identifying patient care needs

Through holistic information gathering (i.e., assessing the patient, listening to patients and their families, and critically evaluating clinical findings), experienced nurses are able to narrow the scope of possible clinical problems the patient may be experiencing and rapidly identify their needs. Due to their extensive experience managing myriad presenting patients, experienced nurses possess substantial knowledge that allows them to quickly identify subtle changes in a patient’s status, and to effectively identify patient care needs.

*Importance of patient assessment*. Experienced nurses recognize the importance of performing a thorough patient assessment and utilizing observational skills to obtain critical patient information.

“I think that’s [observational skills] what makes nurses good nurses. I mean, I tell people, ‘I get paid to be observational.’ That’s what I’m good at, so I’m observing the old-fashioned everything: color, demeanor, motion, breathing… If there’s family there, how are they acting. That’s probably the most important. I don’t even necessarily have to have the numbers.” (N2)

“I’m going to obviously look at the person, look at the skin color, look at the breathing pattern, blood pressure, the pulse rate, listen to the heart rate, check the peripheral pulses, the skin, temperature, the color if there is anything, the anxiety in the person, if they’re short of breath, if they feel short of breath, what their symptoms are, what the position of the bed is, what exactly they’re doing, what medications they’re on, what kind of disease processes do they have that could be part or contributory to what’s going on with them…what kind of things really put them at risk.”(N4)

*Holistic approach to patient assessment*. Beyond physical observations, experienced nurses consider the effects of the patient’s environment outside the care setting.

“As I’m more experienced, they’re [factors contributing to patient risk] going through my mind faster and I’m either accepting or denying whatever that is. What’s that risk, it could be this, it could be that. So, really thinking about all the different scenarios that could be occurring and could be contributory to the deterioration that I see.” (N4)

*Identifying potential risks and considering the big picture*. Experienced nurses also know to expect the unexpected and can use their foundation and experiences to consider the big picture of patient care.

“What are the risks to that person? … If I don’t think about what the risks are, I’m just going to be responding to the next task, to the request, to what’s going on in front of me versus really thinking about it. … That context has to include everything that could possibly happen to that person. Not just what I see, what the monitor says, but pulling all of that together. (N4)

*Establishing relationships with patients and families*. Experienced nurses recognize the importance of the patient’s perspective when obtaining information about their status. The patient and their family often knowingly and unknowingly share clinical information that may relate (i.e., directly or indirectly) to their acute illness or a change in status, which nurses use in their assessment of patients.

“I am a firm believer that you talk to your patient because they are going to tell you everything you need to know. You can have all the diagnostic data in the world, but you’re not going to know anything unless that patient talks to you and tells you how they’re feeling because that directs your entire focus of what to look into.” (N5)

“I would always talk to the family and say, ‘This is your loved one. You know them best. I want to hear how it differs from how they were before. Were they able to tell you what they ate for breakfast today? Is that a change, or what is their baseline… I mean before and after,’ and I even like to put that in my notes so that anybody following can say, ‘This is what their normal is.’” (N1)

#### Workload management

Experienced nurses are able to prioritize and bundle their care tasks based on the needs of their patients. Accordingly, this prioritization of tasks and information allows nurses the ability to retain all critical information, which enhances the effectiveness of their decision-making processes.

*Prioritizing critical information*. Experienced nurses in the present study describe the development of cognitive schema to help them prioritize critical information and pull relevant information from long-term memory to support their decision-making processes.

“When you’re a new nurse, you have a really short stack because you can’t remember it all. When you’re more experienced, you have a bigger stack and it’s not stacked like a stack of pancakes. It’s stacked vertically [so] that if it goes too far back, it’s going to fall off or we’re not going to remember what’s in the stack or your stack’s full and it falls out, it falls totally out and you forget something that’s going to be critical. I think that’s what happens as a nurse. You either are able to stack and control and pull out the important volumes or not.” (N4)

*Bundling care tasks*. Experienced nurses bundle their face-to-face care tasks allowing them to spend more time in rooms with patients. Given the importance nurses place on building rapport with patients and gathering information through listening to information provided by patients and their families, bundling care tasks may allow for more time to potentially observe patient status changes.

“Now I do that with my patients. I do my physical assessment with my patients, and now the rest of day I can think about real things happening with my patients. I’m not cluttered up with these other shenanigans.” (N9)

#### Missed care opportunities inform learning

Experienced nurses seek to learn from their patient experiences through critical reflection of unforeseen events or errors during care interventions, case reviews, medical researching, and debriefing with other members of the care team.

“After every trauma or CPR or anything like that, we always have that debriefing of what we could do better. … I maybe not have seen something that I … or another nurse done [sic] wrong that we can do to prepare us for another patient that comes in. Maybe … I didn’t know how to put the LUCAS on one day, so they showed me a different way that we could put it on for the next patient that comes in. I guess it’s just people telling you, criticism, to make you stronger is all it is. That’s what I like. And learning.” (N3)

“You call it debriefing, I call it a root cause analysis because we got to find out what happened. What was the process break that occurred that allowed these things to happen? So when we have an event, we have to go back through the situation, look at the steps, look at what we currently would do and what were the breaks, what do we have to fix so that this doesn’t happen the next time.” (N4)

### Impact of COVID on patient care and nurse decision-making

**Fig 3:**
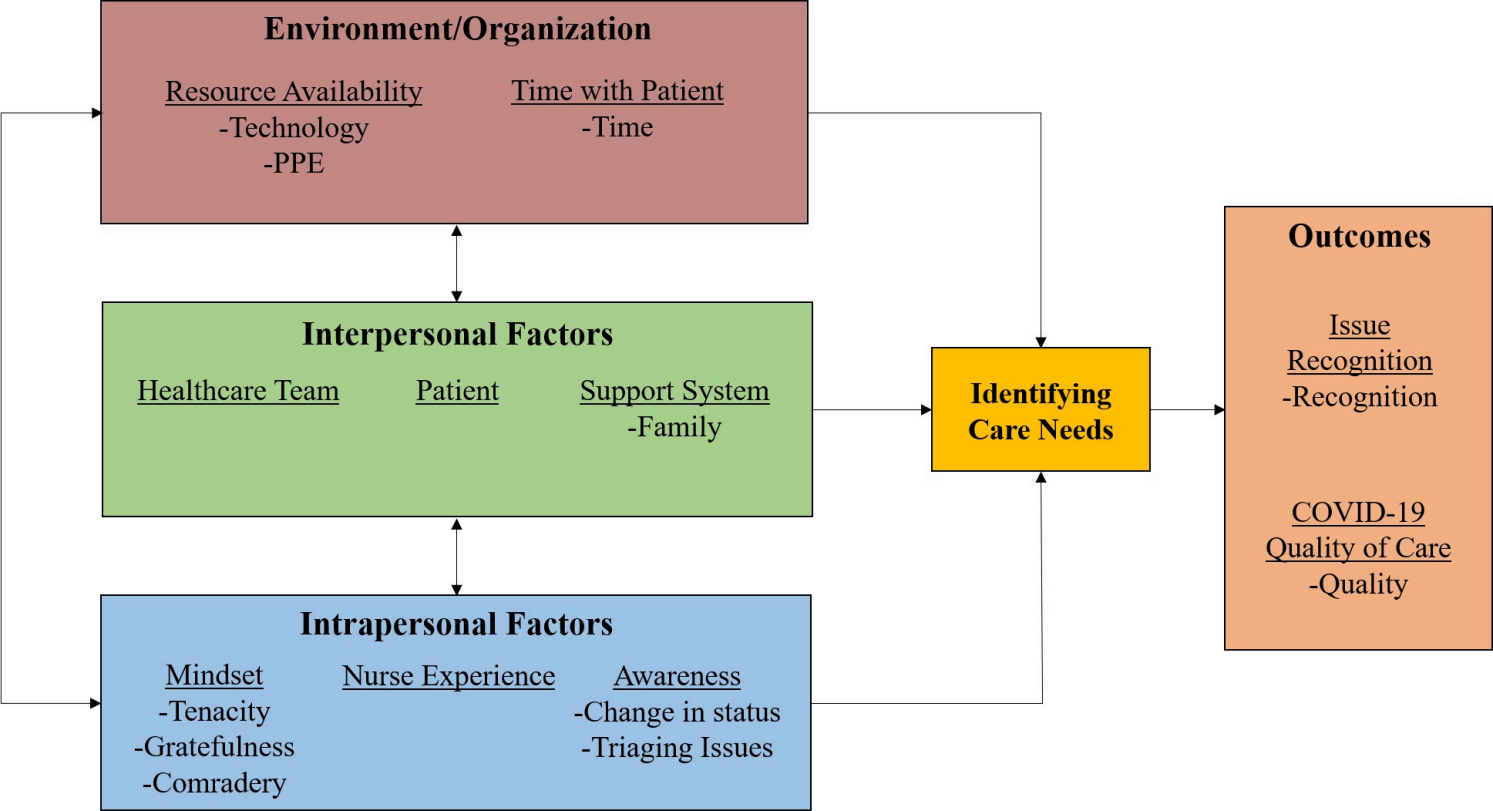
Impact of COVID-19 on nursing care processes and care delivery.

The rapid emergence of COVID-19 has had wide-ranging effects on nurses’ ability to deliver patient care. The effects of the pandemic on decision-making reported by experienced nurses ranged from the individual or intrapersonal factors (e.g., maintaining awareness for rapidly changing patient status), the interpersonal level (e.g., the inability to gather information from patient families), and to the systems level (e.g., availability of personal protective equipment (PPE) to facilitate direct contact with patients). Findings are summarized as a work system model (Fig 3) by adapting the Systems Engineering Initiative for Patient Safety (SEIPS) model [22, 23]. The original model posits that multiple work-system elements (i.e., people, environment, tasks, technology/tools, and organizational factors) interact and influence healthcare processes.

Importantly, we have adapted the SEIPS model to focus on key aspects relevant to nurse decision-making that emerged through our thematic analysis. Specifically, we divided the “people” domain into interpersonal and intrapersonal factors, and have focused the “processes” on “identifying care needs”, as we feel this better captures the impact of different domains on nurses’ decision-making, and ultimately, their ability to identify care needs for patients.

#### Environment/Organization

This theme focuses on key macro organizational effects of COVID-19 on nurses’ ability to deliver safe, quality care. Specifically, it describes organizational resources that affect nurses’ ability to make effective and timely decisions. These factors include things such as physical resources (e.g., PPE) that enable interactions with infected patients and the ability to deliver care in the clinical environment, as well as time resources to sufficiently identify care needs and treat patients.

Resource availability refers to the availability of key resources, such as technology (e.g., respiratory ventilators), PPE (e.g., N-95 masks), and staff that has changed as a result of the high prevalence of COVID-19 and the high demand for these resources. Experienced nurses find that greater resource availability enhances their decision-making ability, as nurses are able to spend more time with their patients if they do not need to strictly ration PPE, they can recognize potential patient complications or deterioration if technology is available, and additional staff coverage can compensate for staff who contract COVID-19. Unfortunately, due to the abrupt onset of pandemics, PPE is extremely scarce. Nurses now must choose between conservation of supplies and patient monitoring which can potentially reduce their ability to administer care.

“That’s a problem, even now with our COVID units, because you know, the national is like, ‘Conserve PPE, conserve PPE, don’t go in your rooms, spend all your tasks together.’ Well, while that’s true, that doesn’t mean that that supersedes my monitoring of the patient. And so I’m constantly struggling, I’m looking at the patients every day over there saying, ‘Why is there a four hour gap between vitals? Don’t you think that we need to go in there and document what the respirations were like earlier? … [Why] are we waiting another four hours to check him again?’” (N10)

Time spent with patients is critical for nurse decision-making as it affords nurses more opportunities to observe changes and collect information, which informs their ability to identify potential issues and respond. A surprising finding on the impact of COVID-19 is on time resources. Prior to the pandemic, time spent interacting with families represented a significant time allocation for nurses on care delivery. Since the onset of COVID-19 and the elimination of family visitors, nurses may experience more time availability to directly interact with patients, as additional time is needed to observe changes and recognize health abnormalities in COVID-19-infected patients.

“Being with a patient face to face, I can assess them no matter what I’m doing. Just being person to person, it’s an important thing. … [whereas] if I was running errands for families and doing this and that, [it] would take it away from patient care.” (N2)

However, there is also the possibility that due to COVID-19, nurses must spend more time donning and doffing PPE and following COVID-19-related protocols than before. This time requirement could ultimately offset the additional time afforded by restrictions to family and visitors that would normally demand nurses’ attention.

“So it’s [PPE] exhausting like I would say physically and emotionally just exhausting in regards to what they go through and so that [PPE Protocol] burns them out, you know, especially when you’re doing all these extra tasks and when you add all these tasks to nurses that’s less time they get to actually spend with the patients.” (N1)]

Technology is a resource whose availability can significantly impact the quality of care. The integration of various physiological sensors and monitors into the electronic medical record (EMR) can allow for automatic charting and trending data to be extracted by nurses, which can give them in-depth insight into how a patient’s status has evolved since their arrival at the hospital. This is particularly helpful to document trends in COVID patient status.

“It’s helpful for me because I can get into the charts and I can look … at the trending graphs … and I can easily see … with all the patients on the COVID unit … what’s their trend for their temperature what’s the trend for the respiratory rate, what’s their trend for their heart rate?” (N10)

#### Interpersonal factors

Interpersonal factors refer to the key individuals or groups of people who are involved in the COVID-19 care process mentioned by experienced nurses in the interviews. The healthcare team consists of providers who care for patients, specifically nurses and physicians. Under normal conditions and depending on the patient’s situation, they may have a support system consisting of family members present throughout their stay in the hospital. As a result of COVID-19, families are unable to be in the healthcare facility with the patient. Thus, families play a smaller role in the care process than usual. Although this is a change due to necessity and not choice, the nurses in this study find that the absence of family members in the COVID-19 restricted care setting prevents nurses from having an additional source of information about potential changes in patient status. Additionally, the lack of family presence can also result in patients experiencing negative emotional states.

“We can’t have any family or visitors, and so I feel like that impacts my patients. Like some of them get very disoriented and their mood can be very low because they don’t get to see their family members.” (N1)

The heightened workload associated with COVID-19 has also put a strain on staff interpersonal relationships. For nurses in leadership positions, they must be mindful about acknowledging the contributions of team members.

“I had a CNA [Certified Nursing Assistant] tell me that she didn’t think I was listening to her and I had to explain to her, I heard you and it’s on my list, but I have to prioritize my entire day. Please don’t ever think I’m not listening or I didn’t put you down on my list. They need to feel like you’ve heard them when they come to you. That’s an important part of the relationship too because they are your eyes.” (N2)

#### Intrapersonal factors

Intrapersonal factors refer to key psychological or experiential traits of persons that can ultimately contribute to clinical care outcomes. Experienced nurses highlight the importance of situation awareness and close monitoring of patient status, including vital signs and behavior, particularly during COVID-19, as patient status can change rapidly.

“Nurses really need to be looking for the monitoring and seeing that changing in the trending to recognize those things to prevent failure to recognize and failure to rescue. And that goes for all patients, but especially to me in this COVID situation that we have right now. … looking ahead like, I need all their vital signs, I need their oxygenation saturation so I can see who we really need to focus on.” (N1)

Nurses report that despite the stressful care environment that COVID-19 presents, their care teams maintain a more determined and grateful mindset and an overall comradery with other members of the care team that helps them maintain a positive disposition, accomplish the tasks required for effective patient care, and enable them to more easily recognize patient issues.

“What I found to be the most impactful with the nurses and the staff working on those COVID floors when they cared for these patients, they didn’t let their fear get in the way. … I said, ‘You know, it’s pretty incredible. The morale here is so high. It’s so good,’ and one of the nurses said, ‘Yeah. I’ve not heard anybody complain once. Everyone is just so thankful to be here, to be alive,’ and I just thought that was a really neat and unique experience that I’ve never felt before. … It definitely impacted our patient care.” (N5)

However, not all healthcare environments are positive. In situations and settings where there is failure or extreme exhaustion when caring for COVID-19 patients and dealing with protocols related to COVID-19 (e.g., PPE), nurse morale suffers. Therefore, it is likely that patient care can be compromised due to disengagement.

“I feel like the PPE is very exhausting, so I feel like it creates a negative impact…I feel like the majority [of nurses] go in trying to do the best they can do that day. When you throw all these extra tasks and things and give them all these other things that they need to be doing to complete their work for that day, if they don’t get that done they feel very defeated…if you throw in a patient who possibly wasn’t on their radar and declines and they miss something, you know that also makes them feel very defeated. I feel it [COVID-19] has definitely impacted nursing emotionally.” (N1)

## Discussion

The goals of the present study were to identify what elements contribute to experienced nurse decision-making when caring for deteriorating patients and explore how the COVID-19 pandemic has impacted nurse decision-making. In summary, we found that experienced nurses are cognizant of the importance of conducting a thorough patient assessment and consulting trends in patients’ health status that are documented in the EMR, but also consider factors in their patients’ environment (i.e., socio-economic status, living situation, etc.) that could be impacting their health and presenting illness. Furthermore, experienced nurses gather information from communicating with patients and their families. All of these factors help nurses maintain a perspective on the “big picture” and identify risks to their patients’ health based on all of the aforementioned factors.

The experienced nurses in this study referenced their ability to systematically work through this multitude of factors quickly, and utilize critical thinking to accept or deny information based on its credibility or pertinence. This finding is supported by the literature on critical thinking in nurse decision-making, as the use of critical thinking for data verification and analysis is an important cognitive skill for nurse effectiveness [13, 14]. Furthermore, participating nurses reported that, through experience, they have learned to rapidly process this information to consider the potential reasons for patient deterioration. While critical thinking is assuredly a component of this analysis, it is likely combined with experience-derived intuition to rapidly rule out reasons for patient deterioration in the assessment process. Utilizing the Cognitive Continuum Theory [15], we can better understand that experienced nurses likely use a combination of critical thinking and intuition in their decision-making processes.

Experienced nurses also highlighted the importance of workload management in their ability to make effective clinical decisions. Working memory is a cognitive system responsible for the temporary storage of information used in complex cognitive actions including learning, reasoning, and comprehension [24]. However, this system has finite capacity limits. Cognitive load refers to the demands imposed on working memory, which, if excessive, can exceed an individual’s capacity and lead to undesired consequences [25]. For example, cognitive overload can reduce an individual’s sensitivity to task-relevant information, slow their decision-making, and reduce their capacity to attend to task-relevant verbal information [26, 27]. Nurses are faced with inherently high cognitive load in their work, and experienced nurses in this study reported utilizing cognitive schema to “stack” and prioritize critical patient care information, which effectively transitions information from working memory to long-term memory and allows them to incorporate large amounts of information in their decision-making process. Furthermore, cognitive schema also allows experienced nurses to pull relevant information from their long-term memory to help make effective decisions. Conversely, novice nurses may not have developed cognitive schema to manage patients due to their inexperience, which leads to inhibited working memory and high cognitive load in the clinical environment [28]. Helping nurses manage their workload effectively is paramount for educators, as high cognitive load can lead nurses to a failure in situation awareness and recognition of deteriorating patients in a timely manner [29]. However, due to COVID-19, nurses are currently being forced to significantly adapt their practice, and it is important to consider the potentially deleterious effects pandemics can have on their decision-making.

Preventing patient families from being in hospitals due to COVID presents a double-edged sword to nurses. While nurses are not being asked to “run errands” for families, which can be time-consuming, they are losing a vital source of information to inform their decision-making. We also found that PPE restrictions were limiting the amount of face-to-face contact they were able to have with patients, which again represents the loss of a vital source of patient information. Failure to recognize deteriorating patients has been highlighted in the literature as one of the leading causes of poor patient outcomes when they are hospitalized, and leads to significantly higher morbidity and mortality due to delays in provision of timely and appropriate care [1–5]. This is particularly true during pandemics, as the experienced nurses interviewed in the current study highlighted the importance of recognizing rapidly deteriorating COVID patients in being able to effectively treat them. That being said, if nurses are limited in their ability to have direct contact with patients due to PPE restrictions or losing families as a source of patient data, they may miss important markers of patient status changes and fail to recognize patient deterioration.

It is clear that the demands on nurses in response to pandemics are heightened. Nurses are required to care for critically sick patients that could rapidly deteriorate [30]. Therefore, nurses can easily experience heightened stress, fatigue due to staff constraints (i.e., other nurses becoming ill and not being able to work, and a high number of patients per nurse), and even burnout. These factors could, in turn, negatively impact nurses’ awareness and vigilance of patient status, which has been shown to negatively impact nurses’ ability to identify critical status changes and appropriately escalate care protocols [31]. Also, due to the unique clinical challenges that caring for COVID-19 patients can present, even experienced nurses are not able to rely on intuition to inform their decision-making. The Dreyfus model of skill acquisition indicates that as a healthcare provider gains experience, they increasingly rely on intuition to make expeditious clinical decisions [11]. However, given the novelty of COVID-19, experienced nurses may not be able to rely on their previously-developed skills to care for these patients, and they could be susceptible for cognitive overload that can impedes their awareness and decision-making.

There were some limitations with this study. First, our team utilized a purposive sampling approach for study participants, which can potentially bias results due to under-representation of nursing in general, and a lack of generalizability of findings. Our team did aim to conduct snowball sampling and recruit participants who were colleagues of initial participants, but were unknown to the researchers. However, due to the COVID-19 pandemic and the resulting workforce demands for clinical nurses, our team was unable to accrue an adequate sample size relying on snowball sampling only. We were instead forced to rely on a more purposive sampling approach, and several participants were known to the researchers. This approach allowed us to purposely sample very experienced nurses (i.e., 8 of 10 participants had more than 10 years of experience), whose responses may provide valuable insights on the factors that impact nurse decision-making and how nurses have had to adapt due to the COVID-19 pandemic.

Another limitation of this study was our seemingly homogenous study sample. All interviewed nurses were female, and the majority of respondents were between 40–49 years of age with MSN degrees. However, as we previously detailed, it was our intention to recruit experienced nurses to reflect on their clinical experiences caring for deteriorating patients, which is why our study sample is older. Furthermore, the fact that all participants in our study were female is reflective of national nursing employment trends, as the National Sample Survey of Registered Nurses conducted by the United States Health Resources and Services Administration in 2018 found that only 9.6% of registered nurses in the country were male [32]. The participating nurses in our study were diverse, however, in their nursing specialties and work setting (i.e., hospital size), which adds to the generalizability of our findings.

Lastly, we did not solicit participant feedback on emerging themes or their specific responses to questions for clarification, which some may perceive to limit trustworthiness in our data. In regards to the trustworthiness or rigor of our findings, Lincoln and Guba (1985) suggest that in naturalistic studies, there are several techniques that are appropriate to establish validity, reliability, and objectivity [21]. These techniques include member checks, triangulation, and prolonged engagement, among others. According to the authors, member checks, or the process of continuously soliciting reactions of participants to the investigator’s reconstruction of data themes based on responses is the most important technique for establishing trustworthiness [33]. However, Sandelowski (1993) contends that member checks are designed to achieve consensus among participants on key response themes, and any attempt to increase reliability through forced consensus typically leads to diminished validity of findings [34].

Instead of utilizing member checks, our team aimed to establish trustworthiness through triangulation among sources of data and members of our research team when analyzing responses. While typically considered to be a method for establishing validity in a qualitative data set [21], this perspective assumes that triangulation between multiple sources will overcome weaknesses with certain components of the data set [35]. Conversely, our team utilized triangulation to develop a robust sample of responses from diverse nurse participants and multiple perspectives during data analysis. Our participants represented various experience levels, clinical specialties, and work settings, and our research team was constructed of nurse educators with experience in qualitative methodology and clinical education, and human factors engineers with expertise studying DM in healthcare. Thus, we are confident that our triangulation approach has allowed our data to represent multiple viewpoints from the respondent and researcher perspective.

Currently, our team is in the process of obtaining validity evidence of the findings in the current study by studying nurse decision-making in simulated clinical situations. We have recruited experienced nurses to participate in two simulated cases, one being involving caring for patients suffering from COVID-19, and are utilizing objective approaches to study decision-making including eye-tracking and electroencephalogram (EEG). We hope the data obtained in this simulation-based study confirms our findings about the sources of information nurses utilize in decision-making, and the challenges nurses face when caring for patients during pandemics.

## Conclusions

Decision-making themes drawn from our participants’ experiences can assist nurse educators training nursing students on decision-making. An interprofessional partnership between nurse educators, experienced practicing nurses, and bioengineering can develop technology to assist in training novice nurses to manage potential barriers (e.g., resource constraints, lack of family) associated with caring for patients during challenging times prior to encountering care issues in the clinical environment where the patient is ultimately at risk for injury. Nursing practice can utilize these findings to increase awareness among experienced nurses on recognizing how pandemic situations can impact to their decision-making capability.

## Supporting information

S1 Appendix (DOCX)

## References

[1] Ghaferi AA, Dimick JB. Understanding failure to rescue and improving safety culture. Ann Surg. 2015;261:839–40. doi: 10.1097/SLA.0000000000001135 25607758 PMC4385410

[2] Burke JR, Downey C, Almoudaris AM. Failure to rescue deteriorating patients: a systematic review of root causes and improvement strategies. J Patient Saf. 2020. doi: 10.1097/PTS.0000000000000720 32453105

[3] Vincent JL, Einav S, Pearse R, Jaber S, Kranke P, Overdyk FJ, et al. Improving detection of patient deterioration in the general hospital ward environment. Eur J Anaesthesiol. 2018;35:325–33. doi: 10.1097/EJA.0000000000000798 29474347 PMC5902137

[4] Bacon CT. Nurses’ experiences with patients who die from failure to rescue after surgery. J Nurs Scholarsh. 2017;49:303–11. doi: 10.1111/jnu.12294 28384381

[5] Abe T, Komori A, Shiraishi A, et al. Trauma complications and in-hospital mortality: failure-to-rescue. Crit Care. 2020;24:223–36. doi: 10.1186/s13054-020-02951-1 32414401 PMC7226721

[6] Mohammmed Iddrisu S, Hutchinson AF, Sungkar Y, Considine J. Nurses’ role in recognising and responding to clinical deterioration in surgical patients. J Clin Nurs 2018;27:1920–30. doi: 10.1111/jocn.14331 29495093

[7] Fusi-Schmidhauser T, Preston NJ, Keller N, Gamondi C. Conservative management of Covid-19 patients—emergency palliative care in action. J Pain Symptom Manage 2020;60:e27–30. doi: 10.1016/j.jpainsymman.2020.03.030 32276101 PMC7144848

[8] Brier J, Carolyn M, Haverly M, et al. Knowing ‘something is not right’ is beyond intuition: development of a clinical algorithm to enhance surveillance and assist nurses to organise and communicate clinical findings. J Clin Nurs 2015;24:832–43. doi: 10.1111/jocn.12670 25236182

[9] Shinnick MA, Cabrera-Mino C. Predictors of Nursing Clinical Judgment in Simulation. Nurs Educ Perspect. 2021;42:107–9. doi: 10.1097/01.NEP.0000000000000604 32028376

[10] Nibbelink CW, Brewer BB. Decision‐making in nursing practice: An integrative literature review. J Clin Nurs 2018;27:917–28. doi: 10.1111/jocn.14151 29098746 PMC5867219

[11] Benner P, Tanner C, Chesla C. From beginner to expert: gaining a differentiated clinical world in critical care nursing. Adv Nurs Sci 1992;14:13–28. doi: 10.1097/00012272-199203000-00005 1550330

[12] Dreyfus HL, Dreyfus SE. Mind over machine. New York, NY: The Free Press. 1985.

[13] Shoulders B, Follett C, Eason J. Enhancing critical thinking in clinical practice: Implications for critical and acute care nurses. Dimens Crit Care Nurs. 2014;33:207–14. doi: 10.1097/DCC.0000000000000053 24895950

[14] Papathanasiou IV, Kleisiaris CF, Fradelos EC, Kakou K, Kourkouta L. Critical thinking: the development of an essential skill for nursing students. Acta Informatica Medica. 2014;22:283–86. doi: 10.5455/aim.2014.22.283-286 25395733 PMC4216424

[15] Hammond KR. Human judgment and social policy: Irreducible uncertainty, inevitable error, unavoidable injustice. Oxford University Press on Demand; 1996.

[16] Saunders B, Sim J, Kingstone T, et al. Saturation in qualitative research: exploring its conceptualization and operationalization. Qual Quant 2018;52:1893–907. doi: 10.1007/s11135-017-0574-8 29937585 PMC5993836

[17] Kim H, Sefcik JS, Bradway C. Characteristics of qualitative descriptive studies: A systematic review. Res Nurs Health. 2017;40:23–42. doi: 10.1002/nur.21768 27686751 PMC5225027

[18] Bradshaw C, Atkinson S, Doody O. Employing a qualitative description approach in health care research. Glob Qual Nurs Res. 2017;4:1–8. doi: 10.1177/2333393617742282 29204457 PMC5703087

[19] Krippendorff K. Content analysis: An introduction to its methodology. Thousand Oaks, CA: Sage publications; 2018.

[20] Hsieh HF, Shannon SE. Three approaches to qualitative content analysis. Qual Health Res 2005;15:1277–88. doi: 10.1177/1049732305276687 16204405

[21] Lincoln YS, Guba EG. Establishing trustworthiness. In: Naturalistic inquiry. Beverly Hills, CA: Sage Publications; 1985, 289–327.

[22] Carayon PA, Hundt AS, Karsh BT, et al. Work system design for patient safety: the SEIPS model. Qual Saf Health Care 2006;15:i50–8. doi: 10.1136/qshc.2005.015842 17142610 PMC2464868

[23] Holden RJ, Carayon P, Gurses AP, et al. SEIPS 2.0: a human factors framework for studying and improving the work of healthcare professionals and patients. Ergonomics 2013;56:1669–86. doi: 10.1080/00140139.2013.838643 24088063 PMC3835697

[24] Baddeley A. Working memory. Science 1992;255:556–9. doi: 10.1126/science.1736359 1736359

[25] Paas F, Renkl A, Sweller J. Cognitive load theory and instructional design: Recent developments. Educ Psychol 2003;38:1–4. doi: 10.1207/S15326985EP3801_1

[26] Speier C, Valacich JS, Vessey I. The influence of task interruption on individual decision making: An information overload perspective. Decis Sci 1999 Mar;30:337–60. doi: 10.1111/j.1540-5915.1999.tb01613.x

[27] Zanto TP, Gazzaley A. Neural suppression of irrelevant information underlies optimal working memory performance. J Neuroscience 2009;29:3059–66. doi: 10.1523/JNEUROSCI.4621-08.2009 19279242 PMC2704557

[28] Josephsen J. Cognitive load theory and nursing simulation: An integrative review. Clin Simul Nurs 2015;11:259–67. doi: 10.1016/j.ecns.2015.02.004

[29] Koch SH, Weir C, Haar M, et al. Intensive care unit nurses’ information needs and recommendations for integrated displays to improve nurses’ situation awareness. J Am Med Inform Assoc 2012;19:583–90. doi: 10.1136/amiajnl-2011-000678 22437074 PMC3384123

[30] Goh KJ, Kalimuddin S, Chan KS. Rapid progression to acute respiratory distress syndrome: Review of current understanding of critical illness from coronavirus disease 2019 (COVID-19) infection. Ann Acad Med Singapore 2020;49:108–8. 32200400

[31] Scott LD, Rogers AE, Hwang WT, Zhang Y. Effects of critical care nurses’ work hours on vigilance and patients’ safety. Am J Crit Care 2006;15:30–7. 16391312

[32] U.S. Department of Health and Human Services Health Resources and Services Administration Bureau of Health Workforce: National Sample Survey of Registered Nurses. 2018. Retrieved from: https://data.hrsa.gov/DataDownload/NSSRN/GeneralPUF18/nssrn-summary-report.pdf. Accessed: 05/20/2021.

[33] Guba EG, Lincoln YS. Fourth generation evaluation. Newbury Park, CA: Sage Publications; 1989.

[34] Sandelowski M. Rigor or rigor mortis: the problem of rigor in qualitative research. Adv Nurs Sci. 1993;16:1–8. doi: 10.1097/00012272-199312000-00002 8311428

[35] Patton MQ. Enhancing the quality and credibility of qualitative analysis. Health Serv Res. 1999;34:1189–1209. 10591279 PMC1089059

