# Supplementary material for: Identifying factors that nurses consider in the decision-making process related to patient care during the COVID-19 pandemic
Source: PLoS One. 2021 Jul 2;16(7):e0254077. doi: 10.1371/journal.pone.0254077 (PMC8253418; doi:10.1371/journal.pone.0254077)
Supplement: S1 Appendix — (DOCX) [file pone.0254077.s001.docx]

General ice breaker

1. Intro to each other… Most memorable patient (without revealing patient identifiers)

Beginning of interview

1. Let’s talk about a deteriorating patient **Possible probes/follow-up**
   1. What about a patient who was stable and deteriorated?
   2. What about a patient who was crashing or unstable who you stabilized?
2. Comments/definitions of deteriorating patient **clarifying**
   1. What’s most important to you in identifying that patient?
      1. What were you thinking, seeing, doing before, during, after?
   2. What helps you recognize what is going on?
      1. (gut feeling, previous experience, education) **possible probes**
   3. Have you initiated or been part of rapid response? [Note: Moved here after pilot interviews]
3. What has been different during covid? [Note: Added after the first two interviews]
   1. Your decision-making?
   2. Deteriorating Patients?
4. What worked/didn’t work/what would you’ve done differently **possible probes**
   1. What did you learn from the experience you just shared?
   2. What would you do differently/ or what /how would you share with others what you learned?
5. Is there technology that you find helpful or burdensome? When making patient observations?

Is there a “dream” technology that you would create if you could to help you with patient care?

Wrap up Questions

1. What have you learned about yourself when caring for patients? What skills particularly do you bring to your nursing care?
